# Serous Neoplasm of the Pancreas Complicated by Recurrent Pancreatitis and Hemorrhage Due to Pseudoaneurysm

**DOI:** 10.14309/crj.0000000000002012

**Published:** 2026-02-12

**Authors:** Takaaki Shamoto, Hirotaka Suzuki, Hiroshi Matsubara

**Affiliations:** 1 Department of Gastroenterology, Toyohashi Municipal Hospital, Aichi, Japan

**Keywords:** serous neoplasm, acute pancreatitis, pseudoaneurysm, hemorrhage

## Abstract

We present a woman in her 70s who experienced 2 episodes of pancreatitis within 4 months. She was diagnosed with a microcystic-type serous neoplasm 3 years prior. The second acute pancreatitis episode was complicated by intracystic hemorrhage due to a pseudoaneurysm, which was treated successfully with coil embolization. Although large serous neoplasms are associated with complications, a combination of recurrent pancreatitis and intracystic hemorrhage due to pseudoaneurysm is extremely rare.

## INTRODUCTION

Serous neoplasms (SNs) are cystic tumors that commonly occur in the pancreatic body and tail, particularly in middle-aged and older women. With advances in imaging technology, their detection has increased in recent years.^1^ Unlike other cystic pancreatic tumors, such as intraductal papillary mucinous neoplasms, SNs are considered benign with low malignant potential and a favorable prognosis. Therefore, conservative management with regular imaging follow-up is generally recommended once a diagnosis is established.^2^

Although most SNs are asymptomatic, some patients may exhibit abdominal pain. Tumors >4 cm are more probable to cause abdominal discomfort or palpable masses.^3^ In rare cases, SNs might be associated with severe complications such as pancreatitis or hemorrhage. We report an atypical case of SN complicated by recurrent pancreatitis and intracystic hemorrhage due to a pseudoaneurysm.

## CASE REPORT

**Figure 1.**
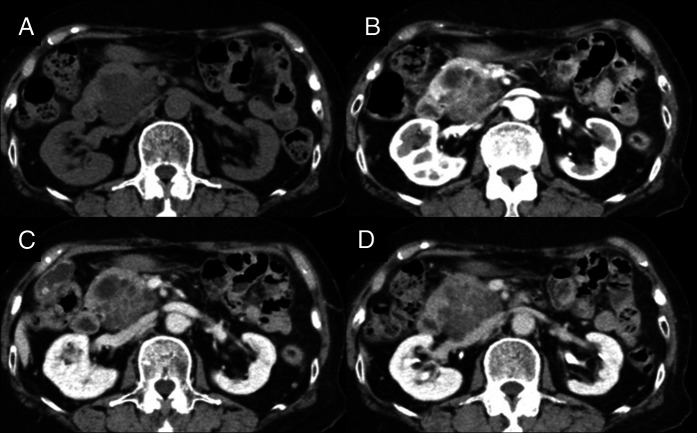
Abdominal dynamic computed tomography. (A) Plain scan, (B) late arterial phase, (C) portal venous phase, and (D) equilibrium phase. A 48 × 45-mm irregular multilocular cystic mass was observed in the pancreatic head. The central area appeared to contain clustered microcysts. The lesion showed maximal enhancement in the arterial phase, followed by washout.

**Figure 2.**
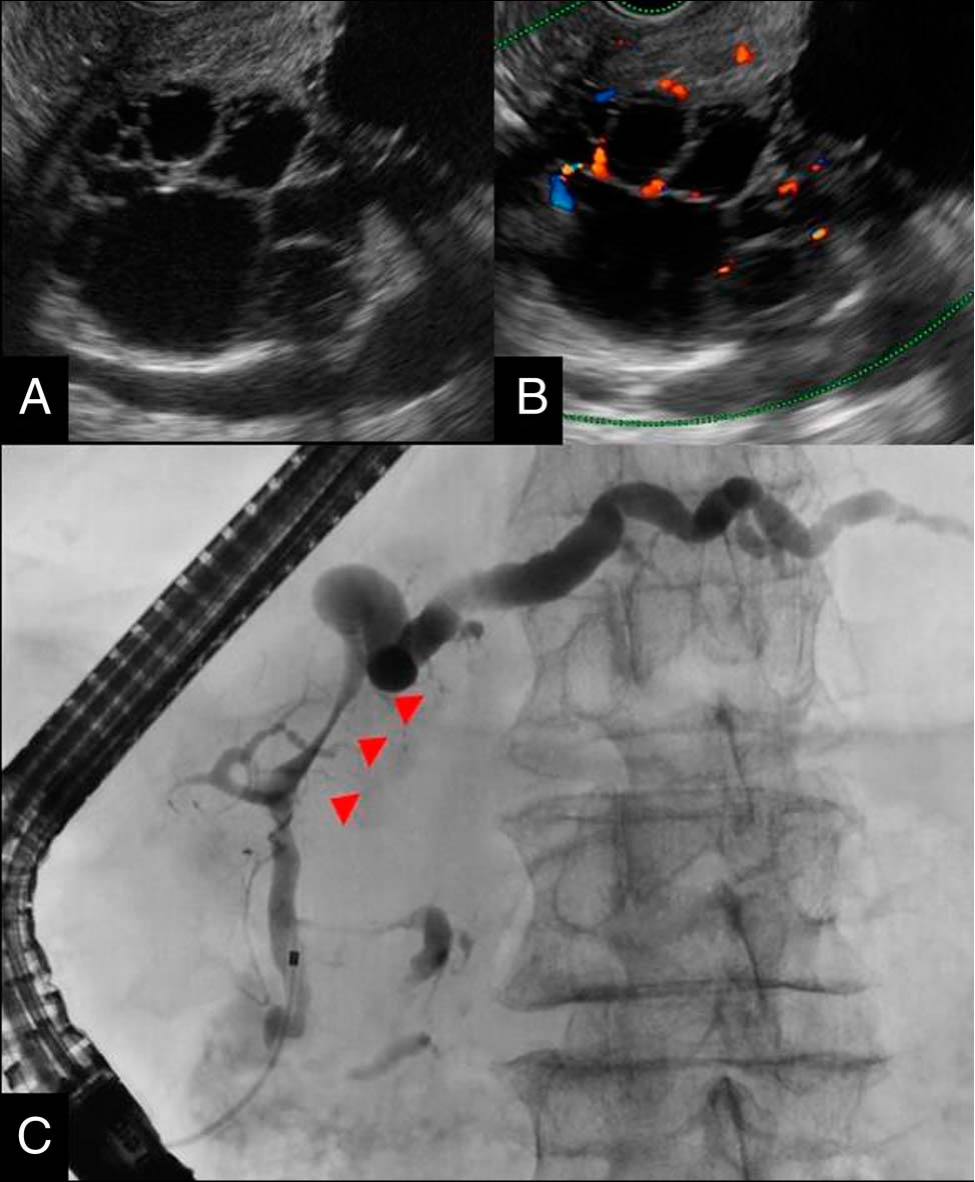
Endoscopic ultrasound. The equipment used was EU-ME2 equipped with a GF-UCT260 echoendoscope. (A) B-mode: honeycomb-like clusters of small cysts were observed in the center of the lesion. No communication with the main pancreatic duct was detected. (B) Color Doppler imaging: blood flow signals were observed in the septa. (C) Endoscopic retrograde cholangiopancreatography. The pancreatic ductogram showed compression of the main pancreatic duct by the cystic lesion (arrowhead) and dilation of the distal duct. No communication between the cyst and the duct was identified.

A woman in her 70s was referred to our department with elevated serum amylase levels. Laboratory test results revealed elevated total amylase (267 U/L) and pancreatic amylase (126 U/L) levels. Contrast-enhanced computed tomography (CT) demonstrated a 48 × 45-mm irregular, multilocular cystic mass in the pancreatic head (Figure 1). Endoscopic ultrasound (EUS) showed a honeycomb-like cluster of small cysts in the lesion center, without communication with the main pancreatic duct (Figure 2). Since the patient had a magnetic resonance imaging–incompatible implantable cardioverter-defibrillator, magnetic resonance cholangiopancreatography could not be performed. To confirm the communication with the main pancreatic duct accurately, endoscopic retrograde cholangiopancreatography (ERCP) was performed. ERCP revealed compression of the main pancreatic duct at the cyst site and dilation of the distal duct. However, there was no communication between the cyst and duct (Figure 2). No complications, including post-ERCP pancreatitis, were observed. Based on these findings, the patient was diagnosed with microcystic-type SN.

**Figure 3.**
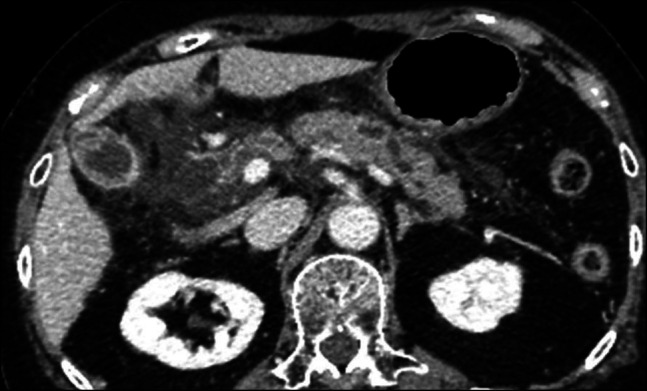
Contrast-enhanced computed tomography. Mild dilation of the distal pancreatic duct, pancreatic swelling, and increased peripancreatic fat stranding were observed and were related to the serous neoplasm.

The patient was followed up with serial CT and EUS, which showed tumor growth (approximately 5 mm/yr) without symptoms. Three years later, the patient presented with epigastric pain. Laboratory tests revealed markedly elevated serum amylase (3,337 U/L) and pancreatic amylase (3,053 U/L) levels. CT identified that the SN was enlarged (63 × 47 mm), with mild dilation of the distal pancreatic duct, pancreatic swelling, and increased fat stranding (Figure 3). The patient was diagnosed with acute pancreatitis and hospitalized. The patient was conservatively managed and discharged 10 days later. Surgery was advised for recurrent pancreatitis; however, the patient declined, and follow-up was continued.

**Figure 4.**
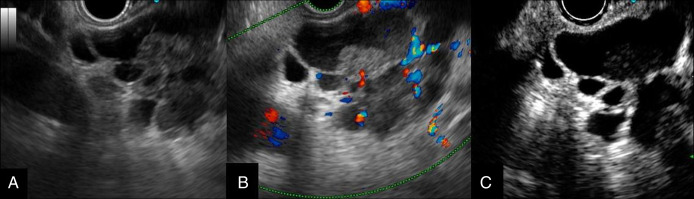
(A) EUS demonstrated a high-echoic area within the cyst. (B) Color Doppler imaging showed no blood flow within the high-echoic area. (C) Contrast-enhanced EUS using Sonazoid revealed no enhancement in the high-echoic area, suggesting intracystic hemorrhage. EUS, endoscopic ultrasound.

**Figure 5.**
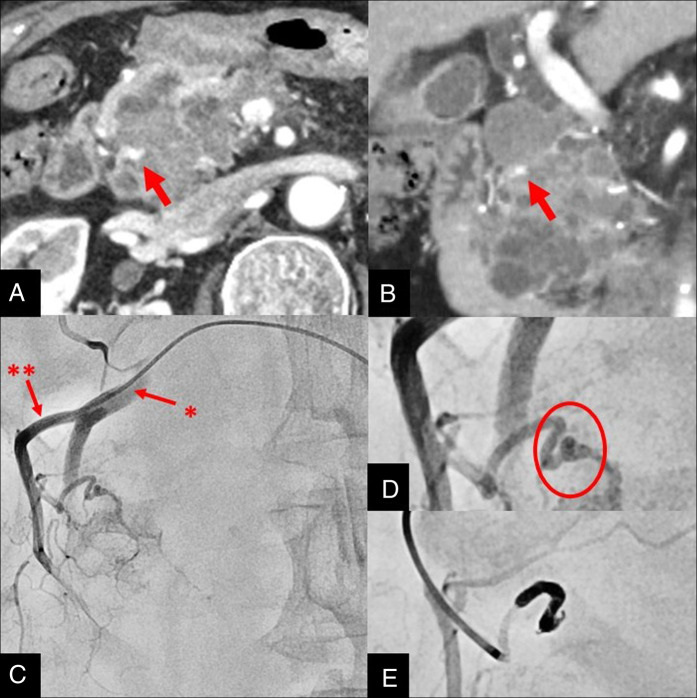
Contrast-enhanced computed tomography. (A) Axial and (B) coronal images demonstrating a pseudoaneurysm within the cyst (arrow). Abdominal angiography. (C) Overview, gastroduodenal artery* and posterior superior pancreaticoduodenal artery**. (D) Magnified images confirming the presence of a pseudoaneurysm. (E) Coil embolization was performed.

Four months later, the patient presented with epigastric pain and was admitted for recurrent acute pancreatitis. Due to the presence of an implantable cardioverter-defibrillator, emergency CT was not performed. Subsequent EUS revealed further enlargement of the SN (>80 mm) and a high-echo area within the cyst, suspecting hematoma. Following informed consent, contrast-enhanced EUS using Sonazoid showed no contrast effect in the high-echo area, suggesting intracystic hemorrhage (Figure 4). Dynamic CT revealed a pseudoaneurysm within the cyst, which had not been observed on previous CT scans (Figure 5). Angiography identified a pseudoaneurysm in the tortuous branch of the posterior superior pancreaticoduodenal artery. Coil embolization was performed (Figure 5); the postoperative course of the patient was uneventful. The patient was discharged after 10 days. Although surgery was suggested, the patient again refused. To date, the patient has remained stable without any recorded recurrence of pancreatitis or hemorrhage.

## DISCUSSION

SNs are typically found in the pancreatic body and tail of middle-aged and older women. The overall malignant potential of SNs is extremely low (<1%); moreover, disease-specific mortality is considered virtually zero.^4^ Therefore, conservative management without surgery is generally accepted once a SN is diagnosed.^2^

Larger tumors (>4 cm) are more probable to cause abdominal pain or palpable masses. Tseng et al proposed surgical intervention for tumors ≥4 cm.^3^ However, Huh et al reported that the average size of serous cystadenocarcinomas was 10.2 ± 4.0 cm, indicating that most malignant SNs are massive.^5^ Galanis et al recommended surgery for SNs that grew by >1 cm within 6–12 months.^6^ Although the criteria for surgery vary, considering resection of symptomatic or enlarged SNs ≥4 cm is justified.

In the present case, although the SN was initially >4 cm, the patient was asymptomatic and thereby was conservatively managed. However, the tumor gradually enlarged and was associated with recurrent pancreatitis. Kimura et al reported that 61% of patients with SNs were asymptomatic, 27% presented with nonspecific abdominal pain, whereas 4% presented with acute pancreatitis.^1^ Kida et al suggested that SN-related pancreatitis may result from tumor-induced compression and narrowing of the main pancreatic duct, which probably occurred in our patient as the SN grew with time.^7^

In addition to pancreatitis, the present case was complicated by intracystic hemorrhage due to a pseudoaneurysm. Possible mechanisms include vascular rupture within the tumor due to progressive enlargement or inflammation involving the vessels around the cyst.^8^ Microcystic SNs are hypervascular, with fibrous septa containing numerous small blood vessels. This characteristic of microcystic SNs might also be related to vascular rupture.^9^ In this case, the initial occurrence of pancreatitis was probably due to ductal compression secondary to tumor enlargement. This, combined with inflammation and tumor growth, might have led to pseudoaneurysm formation. This mechanism of the formation of this pseudoaneurysm is also reinforced by the fact that no pseudoaneurysm was observed on the CT performed before the pancreatitis onset. Subsequent rupture of the pseudoaneurysm caused intracystic hemorrhage, further increasing the tumor volume and resulting in recurrent pancreatitis.

In previous reports, all similar cases involved tumors ≥4 cm and hemorrhage associated with tumor rupture or growth.^8,10–17^ Orihara et al.’s case documented both pancreatitis and intracystic hemorrhage; however, the presence of a pseudoaneurysm was unconfirmed.^12^ In the present case, we identified a pseudoaneurysm as the cause of both SN-related recurrent pancreatitis and hemorrhage.

Large SNs may cause not only pancreatitis but also life-threatening complications such as hemorrhage due to pseudoaneurysm formation. Hence, careful monitoring is vital for SNs measuring >4 cm.

## DISCLOSURES

Author contributions: All authors contributed equally to this manuscript. T. Shamoto is the article guarantor.

Financial disclosure: None to report.

Informed consent was obtained for this case report.

## ABBREVIATIONS:

CT: computed tomography
ERCP: endoscopic retrograde cholangiopancreatography
EUS: endoscopic ultrasound
SN: serous neoplasm
